# Highly pH-responsive sensor based on amplified spontaneous emission coupled to colorimetry

**DOI:** 10.1038/srep46265

**Published:** 2017-04-07

**Authors:** Qi Zhang, Jose R. Castro Smirnov, Ruidong Xia, Jose M. Pedrosa, Isabel Rodriguez, Juan Cabanillas-Gonzalez, Wei Huang

**Affiliations:** 1 Key Laboratory for Organic Electronics and Information Displays & Institute of Advanced Materials, National Jiangsu Synergistic Innovation Center for Advanced Materials (SICAM), Nanjing University of Posts and Telecommunications, 9 Wenyuan Road, Nanjing 210046, China; 2 Madrid Institute for Advanced Studies, IMDEA Nanociencia, Calle Faraday 9, Ciudad Universitaria de Cantoblanco, 28049, Spain; 3 Department of Physical, Chemical and Natural System, Universidad Pablo de Olavide, Seville, ES 41013, Spain; 4 Institute of Advanced Materials (IAM), Jiangsu-Singapore Joint Research Center for Organic/Bio-Electronics & Information Displays, Nanjing Tech University, 30 South Puzhu Road, Nanjing 211816, China

## Abstract

We demonstrated a simple, directly-readable approach for high resolution pH sensing. The method was based on sharp changes in Amplified Spontaneous Emission (ASE) of a Stilbene 420 (ST) laser dye triggered by the pH-dependent absorption of Bromocresol Green (BG). The ASE threshold of BG:ST solution mixtures exhibited a strong dependence on BG absorption, which was drastically changed by the variations of the pH of BG solution. As a result, ASE on-off or off-on was observed with different pH levels achieved by ammonia doping. By changing the concentration of the BG solution and the BG:ST blend ratio, this approach allowed to detect pH changes with a sensitivity down to 0.05 in the 10–11 pH range.

Determination of pH is of paramount importance in numerous scientific and industrial research areas. This parameter controls many chemical and biological reactions in aqueous medium or gas phase. Its measurement is critical for various applications such as clinical^1^, food^2^,^3^,^4^ or environmental analysis^5^,^6^. Different types of pH sensors, such as indicator reagents or strips, pH electrodes (hydrogen electrode, quinhydrone electrode, antimony electrode, glass electrode), and optical sensors^7^,^8^,^9^ are now commercially available. Among the wide range of pH sensors, optical sensors^5^,^10^,^11^,^12^,^13^,^14^,^15^,^16^,^17^ have gained a growing interest owing to the possibility for miniaturization, their immunity to electrical interference and capability for remote sensing. Absorption-based optical sensors have for instance been developed by matching the absorption of some pH indicators with fluorophores such as QDs embedded in a matrix and detect fluorescence at the wavelengths of pH indicator absorption^18^. Such sensors exhibited large reproducibility over 3 cycles (less than 1% difference) but slow response time (50–70 s). There are examples of colorimetry sensors based on RGB LED displays and multiple colour detectors in combination with a range of different pH indicators achieving a pH resolution in the 0.1 range at best^19^. The responses of such devices are in the 1.2 s with an accuracy of 0.2 pH units. Absorption-based sensors have also been implemented in optical fibres by attaching substrates coated with an indicator in a sol-gel matrix onto a plastic optical fibre^20^. The advantage of this system is that it allows for remote monitoring, nevertheless the response times are relatively slow (20–60 s), probably associated to the rigidity of the matrix. There is a broad literature on the synthesis of novel pH-sensitive fluorophores^21^. In many of these works the accuracy and resolution of the sensors are not reported, being the characterization restricted in many cases to show the principle of operation in a certain pH dynamic range, and their reproducibility^22^. In some cases fluorescent indicators were found to exhibit photobleach leading to a lack of reversibility during the measurements over several cycles^23^. Concerning real world applications the sensing specifications provided by some manufacturers like Ocean Optics, PreSens and CellPhase are variable depending on the applications. In general most of the commercially available systems report pH dynamic ranges in the 5–9 with pH resolution in the 0.01 range and time responses of several seconds (30–210 s)^21^. However, these methods are not directly-readable, i.e. they require a comparison with an accurate reference, resulting in a lower degree of sensitivity. Furthermore, the resolution of the sensors is restricted by the limits of the naked eye and the accumulated error of spectrometers.

Herein, we introduce a novel method of pH sensing based on Amplified Spontaneous Emission (ASE) of the lasing dye Stlibene 420 (ST) and its mixture with bromocresol green (BG). ST is a blue emitting laser dye which has a photoluminescence (PL) peak positioned at 420 nm, whilst BG is a widely used colorimetric pH sensor^3^,^14^,^16^,^17^,^24^,^25^,^26^,^27^,^28^. Solutions based on mixtures of ST and BG exhibit ASE at high density fluence, despite partial photon re-absorption caused by the spectral overlap between ST emission and BG absorption. The ASE pH-sensing principle reported in this paper works as follows. Variations in pH trigger drastic changes in the BG absorption spectrum which are in turn reflected into more (less) re-absorbed photons. This effect causes sharp transitions from ASE emission regime of ST (if pump power is just above ASE threshold) to fluorescence regime with a subsequent magnification of the pH response. Thus, subtle variations in pH lead to ASE on-off or off-on. This sensor responds to pH range from 10 to 11 in a directly-readable manner with a 0.05 pH sensitivity demonstrated. Such pH values are typically found on cosmetics such as dental toothpaste, soaps or detergents.

## Results and Discussion

**Figure 1:**
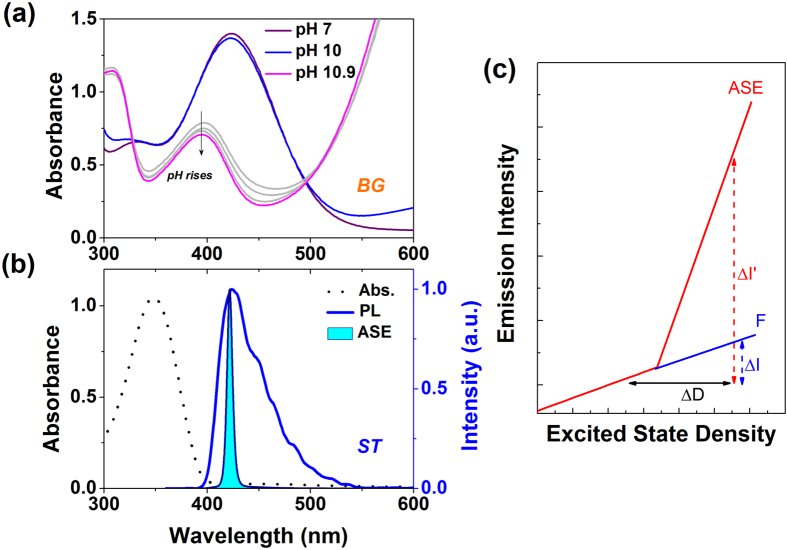
(**a**) Absorption spectra of a neutralized BG solution (0.5 mg/ml in methanol), and same BG solution when different amounts of aqueous ammonia are added (pH ranging from ~10 to ~10.9). (**b**) Absorption spectra of a ST solution (0.1 mg/ml in methanol) (dashed line), photoluminescence (solid line) and ASE (filled area) of ST solution (2 mg/ml in methanol). (**c**) Schematic representation of the ASE sensing principle.

Figure 1(a) shows the absorption spectra of neutralized BG (0.5 mg/ml in methanol) solution and BG solution doped with different aqueous ammonia (20 μl of ammonia solution pH 10 and 10.9, in 600 μl of BG solution). The light gray line represents the absorption of the BG solution when aqueous ammonia dispersions with pH 10.28, 10.53 and 10.78 are added in. The calculation of pH value of the ammonia solution can be found in Supplementary Information (SI). The absorption spectrum of BG changes almost immediately as the alkali volumes are added and it stabilizes at constant level until the alkalinity is neutralized (see Figure S1). Figure 1(b) shows the absorption, photoluminescence and the amplified spontaneous emission spectra of ST (0.1 mg/ml). The absorption of BG in neutral form is characterized by a peak centered at 425 nm, spectrally overlapped with the PL spectra of ST, whereas the absorption of ST peaks at 348 nm. In contrast with ST, BG displays weak absorption at 355 nm (the excitation wavelength employed for ASE measurements). By adding ammonia to the BG solution, the latter turns into its basic form manifested as the emerging of a new absorption band at longer wavelength (magenta line in Fig. 1(a)). The absorbance value at 420 nm (ASE peak position of ST solution) decreased by a 2.9 factor, giving rise to a strong modulation of the ST-ASE in blended solutions, as it will be shown later. Figure 1(c) shows a scheme which illustrates the ASE sensing principle. Our approach benefits from the distinctly superlinear emissive behavior of an optical gain medium optically pumped above the ASE threshold to magnify the pH-fluorescence (F) response. In the ASE regime, changes in excitation density (∆D) lead to a superlinear emission response (∆I’) of significantly larger magnitude respect to the corresponding in the linear regime (∆I). In BG:ST solutions, pH changes lead to ∆D through a combined effect comprising BG color change and more (less) re-absorption of ST emitted photons. The result is a drastic change in ST photon amplification reflected in an increase(decrease) in ASE threshold.

In order to demonstrate this principle and characterize the sensing performance we first develop a method to monitor systematically the pH response upon doping with different ammonia contents. We started with a neutral solution consisting of 600 μl of ST in methanol (5 mg/ml) to which 20 μl of ammonia/water dispersions were added (weight percentages from ~0.003 wt% to ~0.06 wt% corresponding to pH 10.28–10.93). The volume and concentration values were carefully chosen according to two criteria. First, dilution caused by a 3% increase in total volume of the ammonia doped solutions has negligible effect on ASE threshold. This observation was confirmed by performing ASE measurements in ST solutions of three volumes, 2, 5 and 10 mg/ml in methanol, which gave rise to same ASE thresholds and spectra (Figure S2). Second, the total content of ammonia on the final solution was low enough to prevent direct ST-ammonia interactions. From these observations, we deduce that ASE threshold changes caused by ammonia doping must be related to the presence of BG and its characteristic pH-dependent absorption spectrum. In all the BG:ST blend test solutions, the total ST concentration and volume of mixtures was fixed (5 mg/ml and 600 μl respectively), whereas the BG content was changed (1:20, 1:10, 1:7, 1:5, 1:2, 1:1 BG:ST ratios) to tune the ASE behavior.

**Figure 2:**
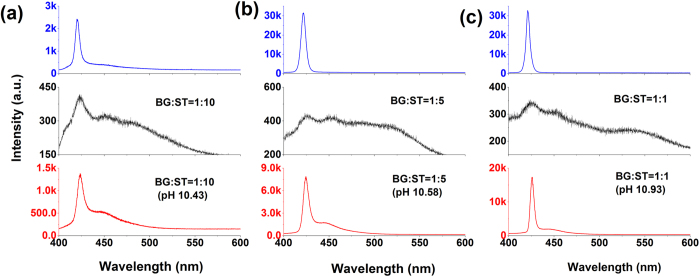
ASE spectra of pristine ST (top), undoped BG:ST solution (middle) and doped BG:ST solution (bottom) for different BG:ST contents: (**a**) 1:10 under 0.94 kW/pulse pumped, (**b**) 1:5 under 2.38 kW/pulse pumped, (**c**) 1:1 under 9.48 kW/pulse pumped.

The sensor sensitivity is addressed in Fig. 2. Figure 2(a) shows the emission spectra of ST (upper), the undoped mixture with BG:ST = 1:10 ratio (middle) and the same mixture doped with pH = 10.43 ammonia dispersion (down) upon excitation with 0.94 kW/pulse. As observed, ST solutions exhibit sharp ASE peak at wavelength of 420 nm when pumped at energy of 0.94 kW/pulse. In contrast, BG:ST blend solutions exhibit a wide PL spectrum under the same pump energy, i.e., the ASE of ST experience “on” to “off” upon adding BG. Note that the absorbance at 355 nm of BG in mixtures is well below that of ST, suggesting that most of the pumped light is absorbed by ST. Therefore ASE changes are more likely related to re-absorption of ST emitted photons rather than absorption changes at the pumping wavelength. Interestingly, the ASE is recovered upon doping with ammonia (pH = 10.43), i.e, from “off” to “on” keeping constant the pump power, due to the BG absorption maximum shifting away from the ST ASE peak at 420 nm. This means, for a fixed BG:ST content, an off-on ASE response will be triggered upon a certain pH value, without need to increase the excitation power.

In Fig. 2(b,c), analogous emission spectra are depicted for two mixtures with 1:5 and 1:1 BG:ST content respectively. Additional results for other BG:ST contents are shown in Figure S3. The direct correlation between the ASE threshold values in ammonia doped solutions and the pH value required to restore ASE can be exploited as a highly sensitive way to read pH.

**Figure 3:**
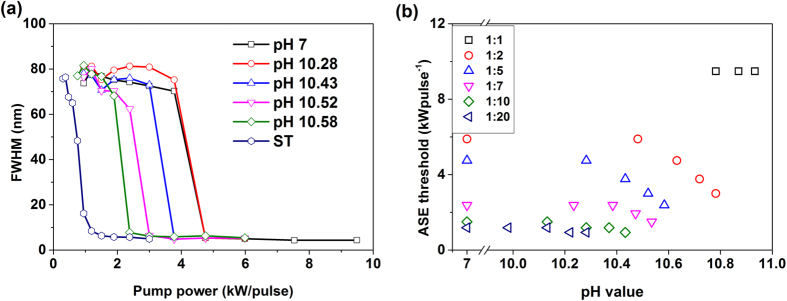
(**a**) FWHM of the emission spectra as a function of the pump power for a 1:5 BG:ST mixture with pH values of 7 (squares), 10.28 (circles) 10.43 (up triangles), 10.52 (down triangles) and 10.58 (diamonds). Pristine ST solution values in neutral conditions are depicted by hexagons. ASE threshold is defined as the pump power when the FWHM decreases to half the value of PL spectra. (**b**) ASE thresholds upon ammonia doping for BG:ST contents ranging from 1:20 to 1:1 (navy left triangle 1:20, olive diamond 1:10, magenta down triangle 1:7, blue up triangle 1:5, red circle 1:2, black square 1:1).

Next, we investigate in detail how the pH values affect ASE. The full width at half maximum (FWHM) values of a 1:5 BG:ST solution are plotted as a function of pump power under different pH in Fig. 3(a). Accordingly, it can be seen that the ASE threshold of the neutral mixture (open square) is a 5.5-fold higher compared to the pristine ST (open hexagon) being their corresponding threshold values 4.75 kW/pulse and 0.94 kW/pulse respectively. Upon increasing the pH value up to 10.28, the threshold of the mixture starts to approach to the corresponding value of pristine ST. It is noteworthy that the sensor is fully recyclable with great stability and reproducibility. By adding proper amount of chlorohydric acid into the ammonia doped BG:ST solution, the ASE threshold of the mixture recover to the same level of the neutral one (see Figure S4 in supporting information).

Then, we characterize the pH sensing capability of different blend solutions with various BG:ST ratios by monitoring ASE threshold upon different levels of ammonia doping, (Fig. 3(b)). Focusing first on mixtures without ammonia, the ASE threshold was found to increase with BG content as expected. Ammonia doping in different amounts (pH 7–11) led to a decrease of ASE thresholds, being the magnitude of this change dependent on BG and ammonia content. In the mixture with 1:20 BG:ST ratio for instance, the undoped ASE threshold was 1.19 kW/pulse. A recovery of the threshold to the level of the pristine ST (~0.94 kW/pulse) was found upon pH = 10.22 ammonia doping. As the BG:ST ratio raised, the amount of ammonia required to restore ASE to the pristine ST level was larger. The ASE threshold of a 1:10 mixture for instance changed from 1.5 kW/pulse undoped to 1.19 kW/pulse upon pH = 10.28 doping, reaching ~0.94 kW/pulse upon pH = 10.43 doping. Finally, for higher BG:ST ratios, it was not possible to recover the threshold to the pristine ST value. The threshold value of a 1:7 mixture changed from 2.38 kW/pulse undoped to a saturated value of 1.5 kW/pulse for ammonia doping above pH 10.53. Similarly, the ASE threshold values in 1:5 (1:2) mixture decreased from 4.75 (5.89) to 2.38 (3) kW/pulse upon pH = 10.58 (pH = 10.78) ammonia doping respectively. Higher BG content (1:1 mixture) is reflected in absence of ASE in undoped solution, even upon pumping with excitation power as high as 30 kW/pulse. ASE reappears however upon 10.78 pH ammonia doping with a threshold value of 9.48 kW/pulse.

**Figure 4:**
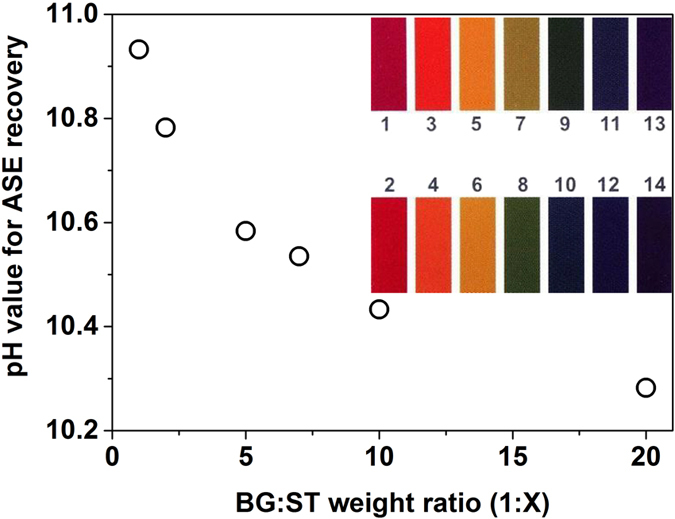
pH values required for ASE recovery as a function of BG concentration in BG:ST blend solutions (pH resolution up to 0.05 ± 0.003). The inset is the standard color chart for the wide range of pH test strips. pH value for ASE recovery stand for the minimum pH required to reach ASE at lowest threshold level in certain mixture. The sensor added value stands on the fact that colorimetric evaluation to the naked eye is no longer feasible in the pH 9 to 14.

In Fig. 4 we recorded the pH values required to reach ASE at lowest threshold level in mixtures as a function of ST ratio against BG (BG:ST = 1:X). We define the resolution of the sensor as the minimum pH change that the sensor can discriminate. From the figure we infer a maximum pH resolution as low as 0.05 ± 0.003 in the 10.28–10.93 sensing range. The resolution of this sensor can be further improved by adjusting the BG:ST ratio. For each BG:ST content the minimum pH value necessary to recover ASE is displayed in Fig. 4 which constitutes the calibration curve of the pH sensor. The protocol to determine the unknown pH of a given sample would consist of screening a set of BG:ST mixtures (i.e. doping BG:ST solutions with a fixed volume of sample and adjust the pump power for different mixtures) and detecting presence/absence of ASE. As the amount of BG in solutions rises, ASE will no longer be feasible for BG contents above a certain BG:ST level, (called here the vanish point). Then, by using the calibration curve displayed in Fig. 4, the pH of the sample will correspond to the one for BG:ST ratio just before the vanish point.

It is also possible to extend the sensing range by using other mixtures which contain different pH-sensitive agents (having different range for pH sensing or absorption spectra) combined with suitable emitters or to tune the initial pH value of the BG:ST solution. For example, if the sample to be sensed is acidic, alkaline buffer can be added to the solution. The main advantage of this sensing scheme is its high resolution. As seen in the inset of Fig. 4, the color of standard pH test strips at 10 and 11 pH values are hardly identified, which hinders the possibility to discern pH within this range using strips. The large sensitivity of the method makes it more interesting for applications in biological media like pH sensing of biological fluids, thus in the 6–8 pH range. For this purpose, laser dyes emitting in near-IR would be more suitable than ST used in this work. Our highly sensitive approach can be extended to such applications providing that there is spectral overlap of dye emission and the pH-indicator absorption in any of its forms. The application of this proof-of-concept for pH sensing in other environments is in scope for future work.

## Conclusion

In conclusion, we have presented a novel highly sensitive pH sensing scheme based on a combination of pH-driven colorimetric response and ASE from a laser dye. This principle allows for pH resolution with sensitivity down to 0.05 pH units. The on/off nature of the sensor response is suitable to neatly identify low pH fluctuations. We investigated the response range of our sensor in 10.28–10.93 by ammonia doping of BG:ST mixtures in order to demonstrate the proof-of-concept. Hitherto, it is possible to extend the sensing range to wider pH ranges by using a suitable buffer solution (with different initial pH value or different mixture of emitter/sensing dye) or other type of pH indicator or emitter. We foresee a high potential of pH sensors based on light amplified emission for future sensing applications.

### Method and setup

All reagents used in this work were of analytical grade and were used without any further purification. All aqueous solutions were prepared with deionized water. The dyes Bromocresol Green (BG) and Stilbene 420 (ST) were acquired from SIGMA-ALDRICH and Exciton, respectively. ST and BG were dissolved in methanol separately. The BG:ST solutions were prepared by blending 1 ml ST solution (10 mg/ml in methanol) to 1 ml BG solution with various concentrations (0.5, 1, 1.5, 2, 5 and 10 mg/ml in methanol) to obtain the required BG:ST blend ratio. In this way, the ST concentration was kept constant (5 mg/ml) while BG concentration changed. This can rule out the dilution effect on the ASE behavior of ST. The ammonia solution (32% w/w) was diluted in deionized water to obtain the required attenuated concentration. The volume of the BG:ST blend solution was always kept 30 times of the volume of the ammonia solution, i.e., 600 μl BG:ST for 20 μl ammonia solution added in. Therefore, a slightly concentration change of the BG:ST (caused by added ammonia solution) will raise negligible influence on the ASE performance of ST. All the solutions were contained in quartz cuvettes which had a light path of 1 mm.

Absorption spectra were recorded with a Varian Cary 50 UV-Vis spectrophotometer. For ASE measurements, a Nd:YAG laser (355 nm) (TEEM Photonics) delivering pulses with 300 ps duration at 30 Hz repetition rate was used as pumping source. The pumping light was focused by a cylindrical lens with a focus length of 15 cm and cut by an open slit of 4 mm. A set of neutral absorbing filters were used for attenuating the pump. The outcoming light of the samples were collected by two plane-convex spheric lenses and then coupled into a spectrometer (SP2500, Acton Research) equipped with a liquid nitrogen cooled back-illuminated deep depletion CCD (Spec-10:400BR, Princeton Instruments).

## Additional Information

**How to cite this article**: Zhang, Q. *et al*. Highly pH-responsive sensor based on amplified spontaneous emission coupled to colorimetry. *Sci. Rep.* **7**, 46265; doi: 10.1038/srep46265 (2017).

**Publisher's note:** Springer Nature remains neutral with regard to jurisdictional claims in published maps and institutional affiliations.

## Supplementary Material

Supplementary Information
