# Supplementary material for: Highly pH-responsive sensor based on amplified spontaneous emission coupled to colorimetry
Source: Sci Rep. 2017 Apr 7;7:46265. doi: 10.1038/srep46265 (PMC5384246; doi:10.1038/srep46265)
Supplement: Supplementary Information [file srep46265-s1.pdf]

Supplementary Information

Highly pH-responsive sensor based on amplified spontaneous emission coupled to colorimetry

Qi Zhang[a], Jose R. Castro Smirnov[b], Ruidong Xia[a]*, Jose M. Pedrosa[c], Isabel Rodriguez[b], Juan Cabanillas-Gonzalez[b]* and Wei Huang[d]*

a. Key Laboratory for Organic Electronics and Information Displays & Institute of Advanced Materials, National Jiangsu Synergistic Innovation Center for Advanced Materials (SICAM), Nanjing University of Posts and Telecommunications, 9 Wenyuan Road, Nanjing 210046, China.

b. Madrid Institute for Advanced Studies, IMDEA Nanociencia, Calle Faraday 9, Ciudad Universitaria de Cantoblanco, 28049, Spain.

c. Department of Physical, Chemical and Natural System, Universidad Pablo de Olavide, Seville, ES 41013, Spain.

d. Institute of Advanced Materials (IAM), Jiangsu-Singapore Joint Research Center for Organic/Bio-Electronics & Information Displays, Nanjing Tech University, 30 South Puzhu Road, Nanjing 211816, China

*Corresponding authors: R.Xia (iamrdxia@njupt.edu.cn); J. Cabanillas-Gonzalez (juan.cabanillas@imdea.org); W.Huang (iamwhuang@njtech.edu.cn)

Calculation of pH value corresponding to the ammonia

pH value of the ammonia solution is calculated by the equation below:

$$pH = 14 + \log_{10}\sqrt{Kb * \frac{(D*17+(1-D)*18)*1000*D}{18*17}} \qquad (S1)$$

D is the weight fraction of ammonia in ammonia solution. Kb is the ionization equilibrium constant of ammonia in water.

Table S1 pH value of the ammonia solution calculated for different weight fraction of ammonia (w/w %)

weight fraction of ammonia	0.06	0.035	0.014	0.011	0.0072	0.0036
pH value	10.93	10.78	10.58	10.53	10.43	10.28

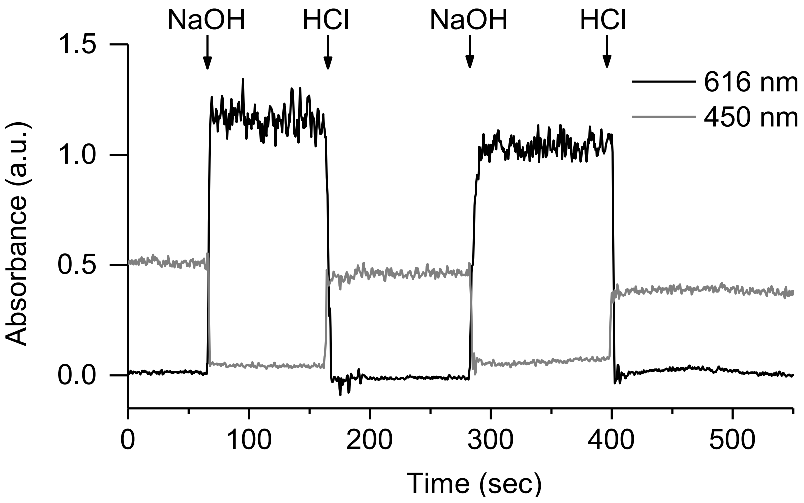

Figure S1. Absorption change of the BG solution at 450 nm and 616 nm as the alkali or acid is added. The edge of the absorbance change is very sharp indicating the response happens fast within the doping. After doping, the absorption stays at the level for at least 100 seconds (can be much longer) in our measurements before neutralization.

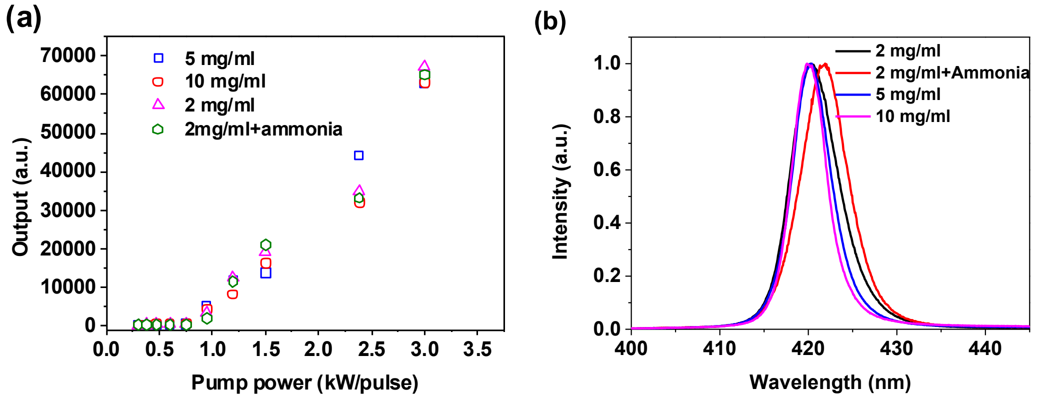

Figure S2. (a) Output intensity of pristine ST solutions with concentrations of 2, 5, 10 mg/ml, together with the 2 mg/ml ST solution with ammonia solution (pH=11.38) added in, are shown as a function of pump power. (b) Typical ASE spectra of pristine ST solutions with concentrations of 2, 5, 10 mg/ml, and the 2 mg/ml ST solution with ammonia solution (pH=11.38) added in.

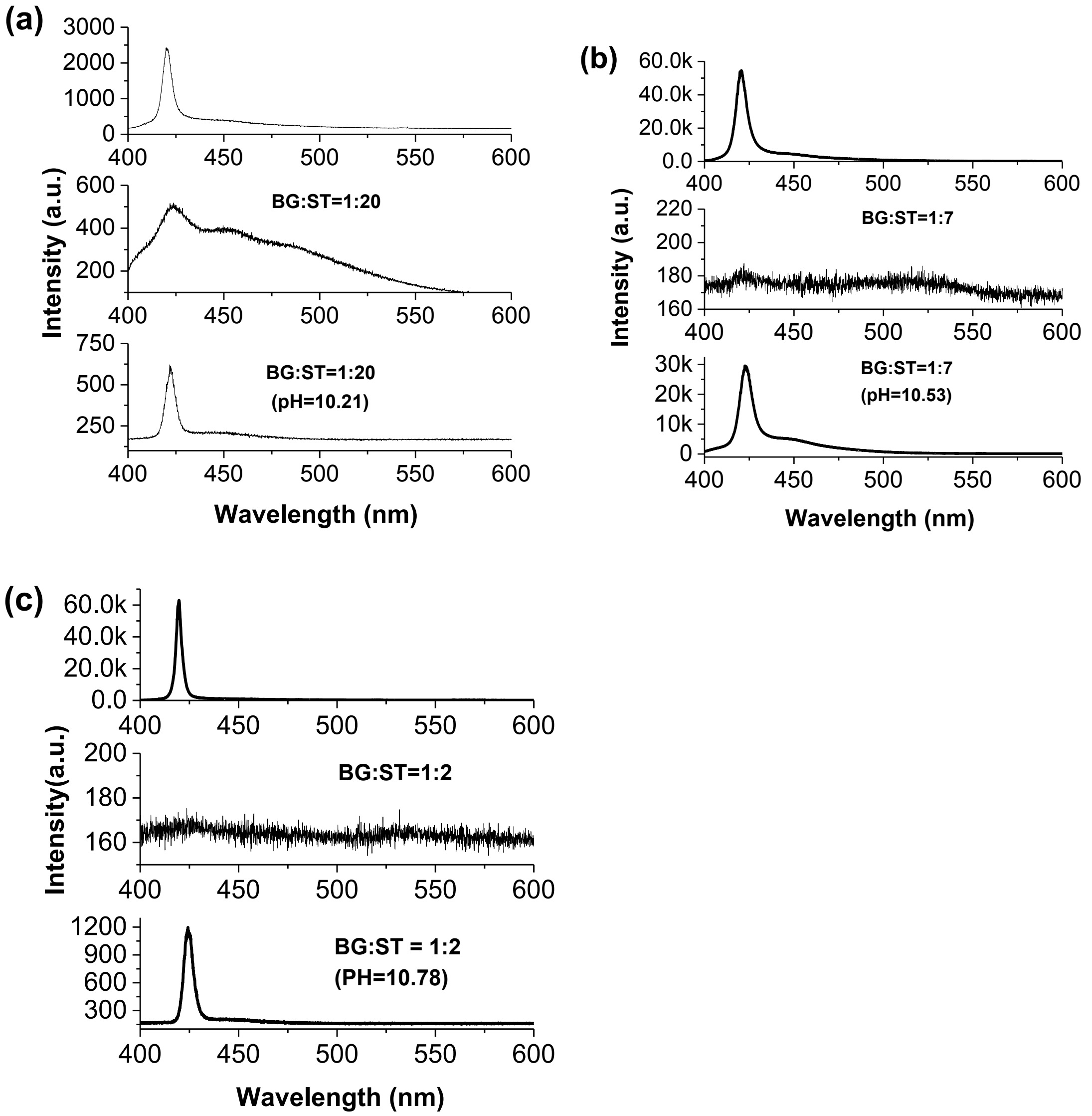

Figure S3. On/off switch of ASE at various BG:ST blend solutions: (a) BG:ST=1:20 under 0.94 kW/pulse pumped, (b) BG:ST=1:7 under 1.5 kW/pulse pumped, (c) BG:ST=1:2 under 3 kW/pulse pumped.

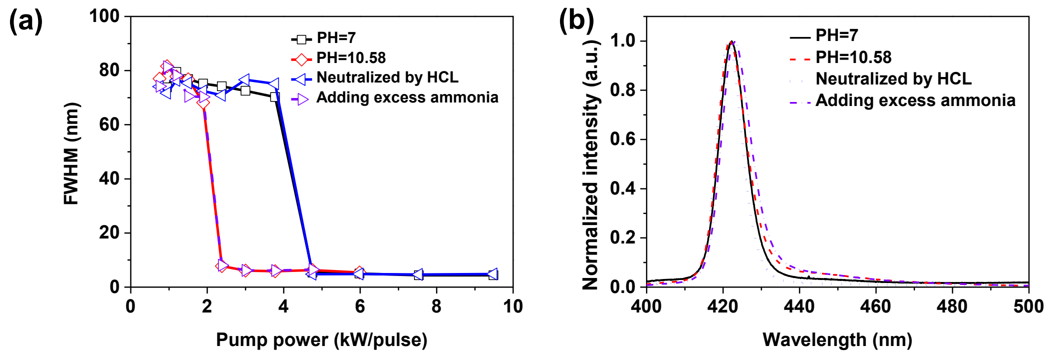

Figure S4. (a) FWHM of the emission spectra as a function of the pump power and (b) ASE spectra for a 1:5 BG:ST mixture undoped (pH=7 squares), doped with pH 10.58 ammonia dispersion (diamonds) and neutralized by chlorohydric acid (left triangles), recycled again by adding excess ammonia (right triangles).

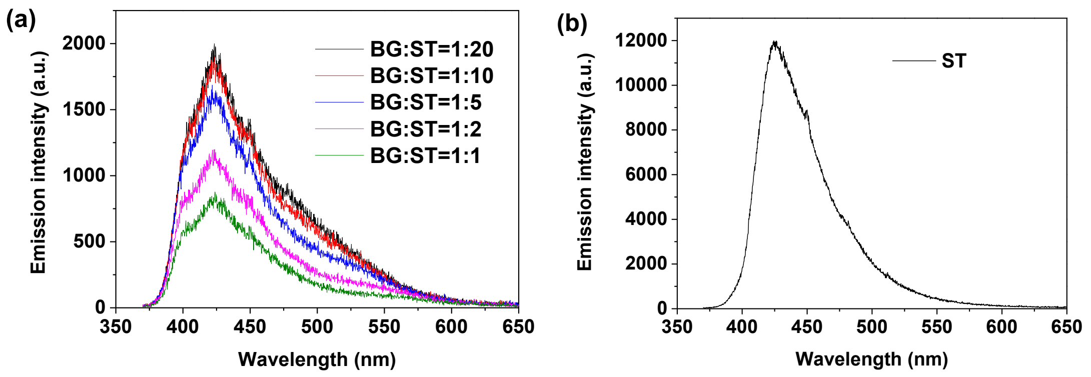

Figure S5. Photoluminescence spectra of the BG:ST blends (a) and pure ST solution (b) measured in an integrating sphere.
